# Non‐Edible Plant Pelemir Seeds (*Cephalaria Syriaca L*.): A New and Effective Source for Biodiesel Feedstock

**DOI:** 10.1002/gch2.70138

**Published:** 2026-08-21

**Authors:** Hülya Karabas, Mahnaz Gümrükcüoglu Yigit

**Affiliations:** ^1^ Department of Environmental Engineering Sakarya University Sakarya Turkey

**Keywords:** biodiesel properties, non‐edible oil, pelemir plant, transesterification

## Abstract

Fossil fuels, as non‐renewable energy sources, pose environmental and energy supply security challenges. This study evaluates pelemir (*Cephalaria syriaca L*.) seed oil, a non‐edible vegetable oil not preferred by the food sector, as a renewable, locally adaptable feedstock for biodiesel production in Türkiye. Unlike edible oils that raise food‐safety concerns, non‐edible oils like pelemir offer a potentially more economical and environmentally friendly route to biodiesel. Crude oil was extracted from Karahan genotype pelemir seeds collected in the Erzincan region. Analyses showed the oil to be rich in unsaturated fatty acids (74.2%), predominantly linoleic and oleic acids. Owing to its low free fatty acid content (<1%), the crude oil was converted into biodiesel by base‐catalyzed transesterification with methanol. Biodiesel produced from Karahan genotype pelemir seeds is suitable for Turkey's geographical and climatic conditions and meets the EN 14214 and ASTM D 6751 biodiesel standards. Feedstock availability and production cost are two key prerequisites for sustainable, commercial biofuel. The use of the pelemir plant as a biodiesel feedstock supports energy security and resilience, as well as emission control for the climate crisis, both globally and in Türkiye.

## Graphical abstract

This research explores sustainable biodiesel production from non‐edible pelemir (Cephalaria syriaca) seed oil, a renewable energy feedstock. Optimized transesterification yields biodiesel with high conversion and desirable fuel properties, meeting international standards. This study highlights pelemir's significant potential for green energy solutions, particularly for regions with similar geographical and climatic conditions to Türkiye, reducing reliance on fossil fuels.

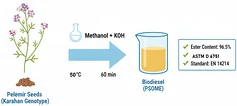

## 1 Introduction

Climate change, caused by global warming, primarily affects agriculture, livestock farming, clean water, and energy resources. Globally, all countries need to diversify their energy sources and increase the use of renewable energies. In doing so, reducing carbon emissions and protecting the environment should be a priority. Scientists have intensified their search for alternative fuel sources due to environmental concerns, fossil fuel depletion, and the rapid increase in energy demand. Utilizing non‐edible feedstocks and waste biomass‐based resources to produce biofuels is crucial for reducing the carbon footprint of the transportation sector. This also promotes more sustainable and efficient resource use [1].

Agriculture is the starting point of the food chain. Biofuels, considered as energy diversification based on agricultural feedstocks, are among the leading agricultural sources of renewable energy. Vegetable and animal oils are the feedstock for biodiesel production, which holds a significant place within biofuels. The lifecycle of biodiesel begins with the agricultural stage of the product necessary for its production. The main factor threatening the sustainability of biodiesel fuel in the world and in our country is the inability to find sufficient oil feedstocks. For a sustainable future, the most realistic and feasible solution is to meet the oil feedstock needs of biodiesel, which we describe as a domestic green fuel, from oils that are not used in the food sector, not cultivation for agricultural purposes, and do not threaten this sector [2, 3].

Biodiesel is an environmentally friendly fuel that can be obtained from renewable biomass sources, both plant and animal. Globally, over 350 sources of oil feedstock that can be used for biodiesel production are listed in the literature. This high number of feedstock sources makes biodiesel production more attractive. More than 95% of biodiesel production currently comes from edible oils [4]. The use of edible oils for biodiesel production could lead to an imbalance in the global food supply, which in turn could increase global food insecurity [5, 6]. The large‐scale expansion of edible oil crops for biofuel production is caused deforestation and ecosystem destruction.

The properties of biodiesel depend on the characteristics of the feedstock. Many different sources are used as feedstocks in biodiesel production, including canola oil, rapeseed oil, coconut oil, palm oil, soybean oil, sunflower, neem kernel oil, papaya, safflower oil, tobacco seed oil, acorn kernel oil, jojoba oil, rubber seed oil, karanja seed oil, linseed oil, peanut oil, jatropha oil, other vegetable oils, microalgal lipids, used frying oils, and food waste. Many feedstock sources such as oils, animal fats, and sewage sludge are currently used in biodiesel production. Biodiesel, produced from non‐edible plant sources, is environmentally friendly, cost‐effective, and compatible with petroleum‐based diesel fuel. The low cost of producing the feedstock, which constitutes the largest component of biodiesel production costs, directly reduces the overall cost of biodiesel [7, 8, 9].

### 1.1 Why Non‐Edible Oils as a Feedstock for Biodiesel?

Globally, non‐edible oilseed crops can be grown, mostly on fallow lands, potentially reducing deforestation and food supply problems. Non‐edible vegetable oils have been identified as promising biodiesel feedstocks offering a sustainable and economical alternative. Recent studies have identified approximately 77 non‐edible plants whose seeds, fruits, or nuts contain more than 30% oil. These feedstocks have been described as “ideal” for biodiesel production due to their combination of economic, environmental, and adaptability advantages [1, 10, 11, 12, 13, 14]. Four key characteristics are critical in evaluating non‐edible vegetable oils as biodiesel feedstock: high oil content, suitable fatty acid composition, non‐competition in food products, and adaptability to marginal lands and climates. These characteristics make non‐edible oils a promising and sustainable alternative to conventional biodiesel sources [15].

Although oilseed production in Türkiye has shown an upward trend over the years, the current production quantity is insufficient to meet the country's total oilseed needs, and Türkiye meets a large portion of its oilseed requirements through imports. Sunflower is the most widely grown oilseed crop in Türkiye, followed by soybean, cottonseed, rapeseed, sesame, safflower, peanut, poppy, hemp, and flax. Due to Türkiye's geographical conditions and diverse climate and temperature characteristics, all oilseed crops except coconut and palm can be cultivated [16, 17].

Within the scope of the “Basin‐Based Support Model,” which was launched in our country in 2017 with the aim of planned agricultural production, 21 strategic products are supported in 941 agricultural basins, including oilseeds such as sunflower, cotton, canola, safflower, and soybean.

**FIGURE 1:**
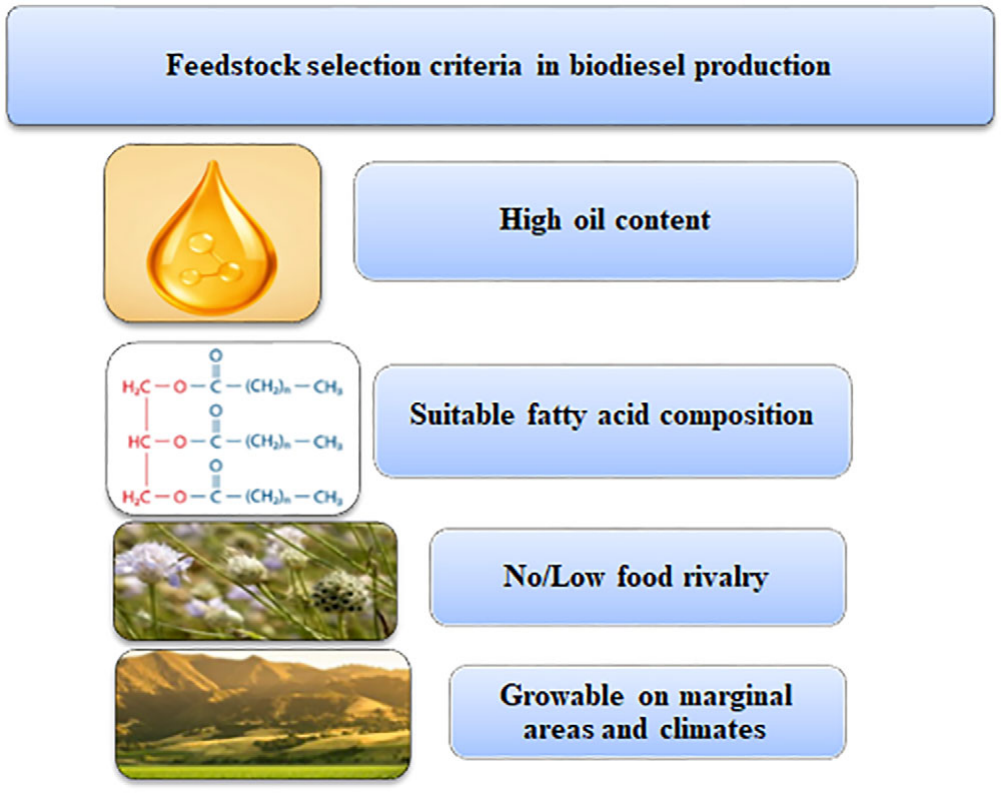
Flowchart showing the process of selecting non‐edible vegetable oil feedstock for biodiesel production.

The flowchart in Figure 1 shows the key criteria for selecting non‐edible vegetable oils to be used as feedstock in biodiesel production.

Our country's geographical location and topographic structure have led to the existence of diverse climate types. Cultivating energy crops by accurately analyzing the regions of our country with diverse ecological conditions is a priority for energy agriculture. Biofuels will be advantageous because they cause less environmental pollution during their use, avoid massive waste generation, and can be used on barren land instead of land used for food agriculture, respectively [18, 19, 20, 21, 22, 23]. In this context, utilizing the Pelemir plant would be advantageous in helping to ensure the supply of oil feedstocks, a fundamental problem in the biodiesel sector that remains relevant both globally and in our country. Biodiesel produced from the oil obtained from the seeds of this plant would be a significant added value for energy agriculture. It is essential to evaluate the pelemir plant, which provides an advantage with its high ecological tolerance, especially in the fallow agricultural areas and in regions with soil and climate conditions suitable for grain cultivation.

### 1.2 Reason for Selecting Pelemir Seeds as Feedstock

The pelemir (*Cephalaria Syriaca L*.) plant is considered a suitable candidate for biodiesel feedstock due to its fundamental characteristics: it does not compete with agricultural products in the food sector, it does not replace them, it has low production inputs (requires no fertilization or irrigation), and it is effortless to produce.

The basic principle of energy supply security is to distribute risk and to choose the least risky source possible. It is precisely at this point that biodiesel produced from pelemir seed oil (PSO) will stand out with its role in supporting energy supply security. Pelemir, a plant found worldwide and with potential as an alternative source of vegetable oilseeds, is a plant belonging to the order Dipsacales of the family Caprifoliaceae of the genus Cephalaria. Various species are found in nature, and the cultivated species is Cephalaria syriaca Schrad [24, 25]. Cephalaria Schrad is known to be distributed particularly in the Mediterranean region, the Balkan Peninsula, southern Ukraine, the Caucasus, Iran, western China, and the Middle East [26]. Although the number of species belonging to the genus Cephalaria Schrad in the literature is 94, Matthews [27] reported that Cephalaria Schrad was represented by 29 species in the flora of Türkiye and the eastern Aegean Islands. Since then, eleven new species and one new subspecies have been described in Türkiye.

Pelemir is an annual, upright‐growing plant with a hollow, strong stem and bluish‐purple flowers. A highly branching plant, pelemir produces seeds at the ends of its main stem and side branches. Branching is important for increasing seed yield per plant. It is quite cold‐resistant, appears as a weed in wheat fields, and does not require special growing conditions or climate requirements, growing well in clay and loamy soils. However, it can be grown even in sloping and erosion‐prone marginal areas where the soil depth is not great, and satisfactory yields can be obtained for these soils. It can grow in almost any climate suitable for wheat cultivation, but it is particularly well‐suited to autumn planting [28, 29, 30, 31, 32]. This plant, which grows to a height of 40–100 cm, can reach depths of up to 120 cm in the soil thanks to its taproot system. The stem of the pelemir plant is strong and hollow inside. A highly branched plant, seeds develop at the tips of the main stem and lateral branches. Branching is an important factor in increasing seed yield per plant. The seeds are small, thin, and elongated. A single bud can produce 12–20 seeds, and therefore, a single plant can yield between 85 and 400 seeds. The thousand‐seed weight varies between 15.0 and 16.29 g [25, 30, 33, 34].

Due to its myristic acid content, pelemir oil is quite suitable for the soap industry [35]. On the other hand, while it is suitable for use in the leather and textile industries due to its epoxy and erucic acid content, it is not suitable for use as edible oil in the food sector [28].

Recently, consumers are increasingly choosing natural products or products containing natural additives. Various cereal seeds or ancient grains such as quinoa, amaranth, and teff, and components derived from them, are examples of this [36]. Pelemir seeds are nutritionally rich, containing 22%–28% oil, 14%–21% protein, 3%–10% ash, and 9%–30% crude fiber. Consequently, they can be used in small amounts as an additive to delay bread staling. For this reason, it is allowed to grow naturally in wheat fields [37]. It is also used as animal feed. From a pharmacological perspective, essential fatty acids in pelemir are not only important nutrients but also compounds that can have positive effects on many diseases. Besides regulating bodily functions such as heart rate, blood pressure, blood clotting, and fertility, they play an important role in boosting the body's immunity [38]. Studies have shown that the saponin compound found in pelemir reduces blood lipids and cancer risk, and is also effective in treating tooth decay and preventing platelet aggregation. Furthermore, it has been emphasized that foods containing saponin can be used in acute lead poisoning [39].

Although the plant was cultivated and its oil was used in a limited area of Türkiye until the 1970s, its introduction into cultivation and its transition from weed to cultivated plant is relatively recent. Selection studies were first initiated in 2009, followed by agronomic studies. In our country, the Karahan genotype pelemir plant was registered as a winter pelemir variety by the Seed Registration and Certification Center (TTSM) on April 11, 2017, and included in the national cultivar list as an oil variety under the name Karahan due to its winter hardiness, high grain and oil yield, and high oil content [25, 40]. In different geographical regions of our country, especially in the last few years, thanks to public and private companies producing biodiesel, farmers have started to plant the Karahan genotype of pelemir plant as a new feedstock source at increasing rates each year.

### 1.3 Objective of the Study

The main objective of the study is to investigate the biodiesel production potential of seed oil obtained from pelemir, a plant that does not compete with vegetable oils used in the food industry as a source of oil raw materials. The synthesis of methyl esters from pelemir oil enables their commercial application in biodiesel production. In the study, non‐edible seed oil of *Cephalaria Sryicaca L* was used to produce biodiesel via a transesterification chemical reaction with a homogeneous catalyst. First, crude pelemir seed oil was obtained from pelemir seeds using a solvent extraction method. The fatty acid composition and physicochemical properties of this oil were analyzed. Due to the low free fatty acid (FFA) content of pelemir seed oil, biodiesel production was carried out using a transesterification method with a basic catalyst. In the final stage, the fuel properties of the produced pelemir seed oil methyl ester (PSOME) were determined. The ultimate aim is to determine the fuel properties of the produced pelemir seed oil methyl ester and to assess its compliance with the international biodiesel standards EN 14214 and ASTM D 6751, thereby demonstrating the feasibility of utilizing this local resource as a sustainable alternative biodiesel feedstock.

In an era where global challenges such as climate change and food insecurity are increasingly prevalent, the pursuit of sustainable energy solutions must be balanced with the need to protect the global food supply. The widespread use of renewable raw materials for biofuel production exacerbates the food‐fuel dilemma, leading to unintended land use and potentially hindering access to food for vulnerable populations worldwide. In this context, identifying raw material alternatives that do not compete with food is crucial. This study addresses this global imperative by focusing on the Karahan genotype, a native pelemir cultivar from Türkiye. Its unique characteristics, such as its ability to adapt to marginal lands unsuitable for traditional food crops and its inedibility due to erucic acid content, make pelemir a strategically suitable biofuel feedstock.

## 2 Materials and Methods

### 2.1 Materials

**FIGURE 2:**
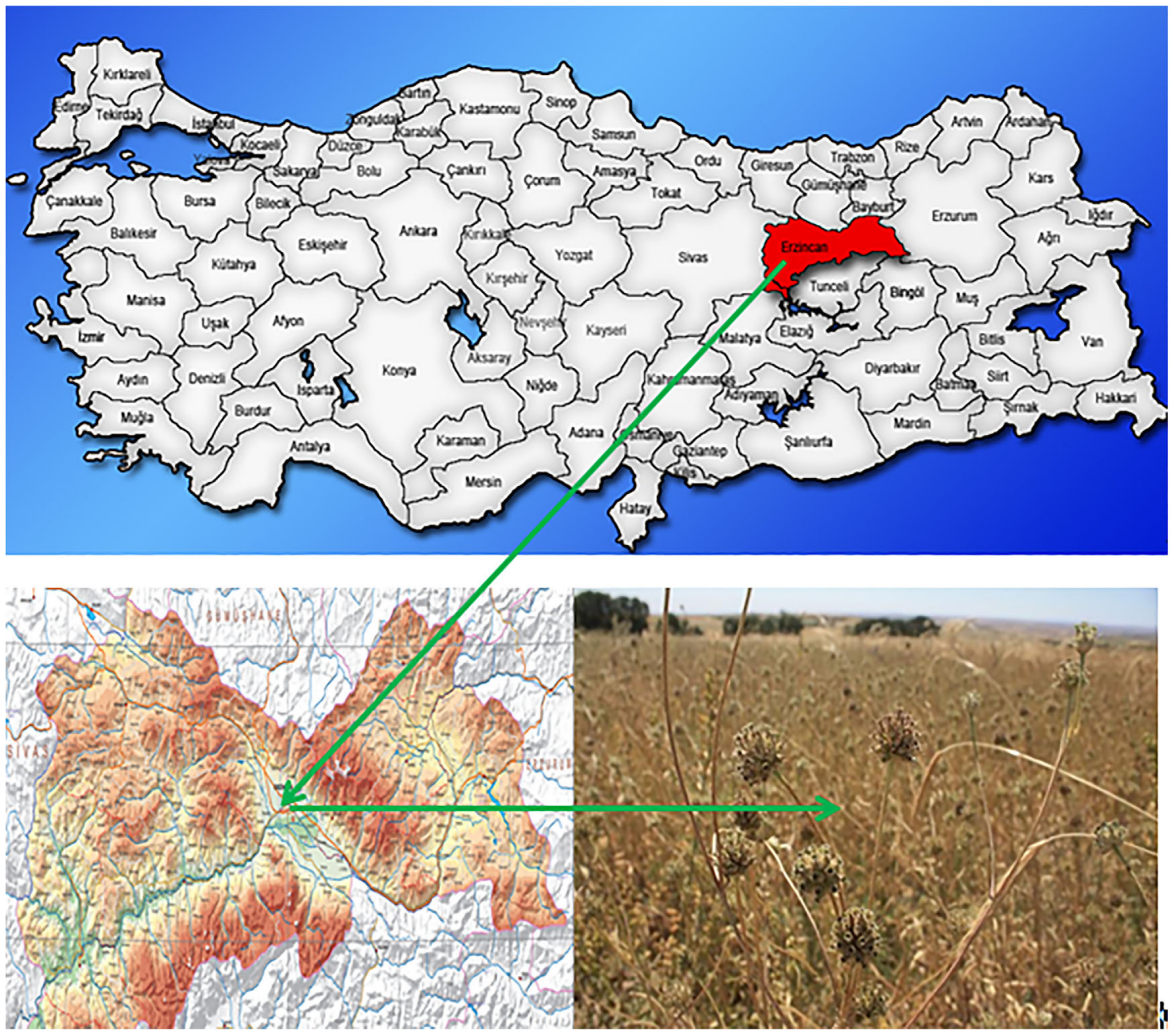
The geographic locations of the harvested Cephalaria Sryicaca **L**. used in this study in Erzincan, Turkey.

In this study, the pelemir plant seeds used for biodiesel production were supplied by a local producer in Erzincan Province, located in the Eastern Anatolia Region of Türkiye. The location of the region is shown in Figure 2.

The pelemir seeds belong to our local variety Karahan genotype, which is the 2023 crop. Oil determination in the seed was carried out using a Gerhardt Soxtherm System ST40 oil analyzer. The density of pelemir oil was determined using the pycnometric method [41]. A moisture analyzer (AND‐MX50) was used to determine moisture in pelemir seed. The oil's color values were determined using a Lovibond brand (L*, a*, b*) color determination device. White and black plates were used for calibration. The L* value represents lightness and darkness, the a* value represents redness and greenness, and the b* value represents yellowness and blueness. Free fatty acidity and acid number were determined using the standard titrimetric method [42]. The refractive index, an indicator of purity, was determined for pelemir oil. An Abbe refractometer was calibrated with water, and then the refractive index of the oil was determined [43]. The peroxide number of pelemir oil was determined by the standard titrimetric method [44]. The Kjeldahl method was used for protein determination, and the nitrogen‐to‐protein conversion factor was used as 5.30 [45]. A waring brand blender was used to grind pelemir seeds. Analytical grade potassium hydroxide (KOH) and methanol were purchased from Merck, Germany. BUCHI rotary vacuum evaporator (Rotavapor R‐210), vacuum pump (V‐700), A D vibro viscometer (SV‐10), and assay balance, HEIDOLPH RZR 2021 mechanical agitator, and NUVE brand NF400 type centrifugal machine were used in laboratory studying.

### 2.2 Methods

#### 2.2.1 Extraction of Pelemir Seed Oil

After cleaning the purchased pelemir seeds from foreign matter. For oil extraction from the seed, pelemir seeds were first ground with a blender and treated twice by mixing with n‐hexane at a ratio of 1:10 for 30 min. At the end of the process, the crude oil was separated using a rotary vacuum evaporator and dried at 40°C. Small particles suspended in the crude oil were separated using a centrifugal machine at the speed of 4200 rpm for 40 min. Total oil content was detected with ‘Gerhardt Soxtherm System ST40’. Physicochemical analyses were applied to the obtained oil.

#### 2.2.2 Transesterification Reaction

Biodiesel is a renewable energy source that serves as an alternative to fossil fuels. It is a biofuel containing a group of long‐chain fatty acids called monoalkyl esters. It is an alternative to diesel fuel produced through a chemical reaction called transesterification of vegetable oils or fats in the presence of alcohol and a catalyst. The main obstacle to using non‐edible oils as biodiesel feedstock is that their high FFA content leads to the formation of emulsions and soaps when reacting with a basic catalyst. Since this reduces the biodiesel production efficiency, the FFA concentration must be reduced before the transesterification reaction.

**FIGURE 3:**
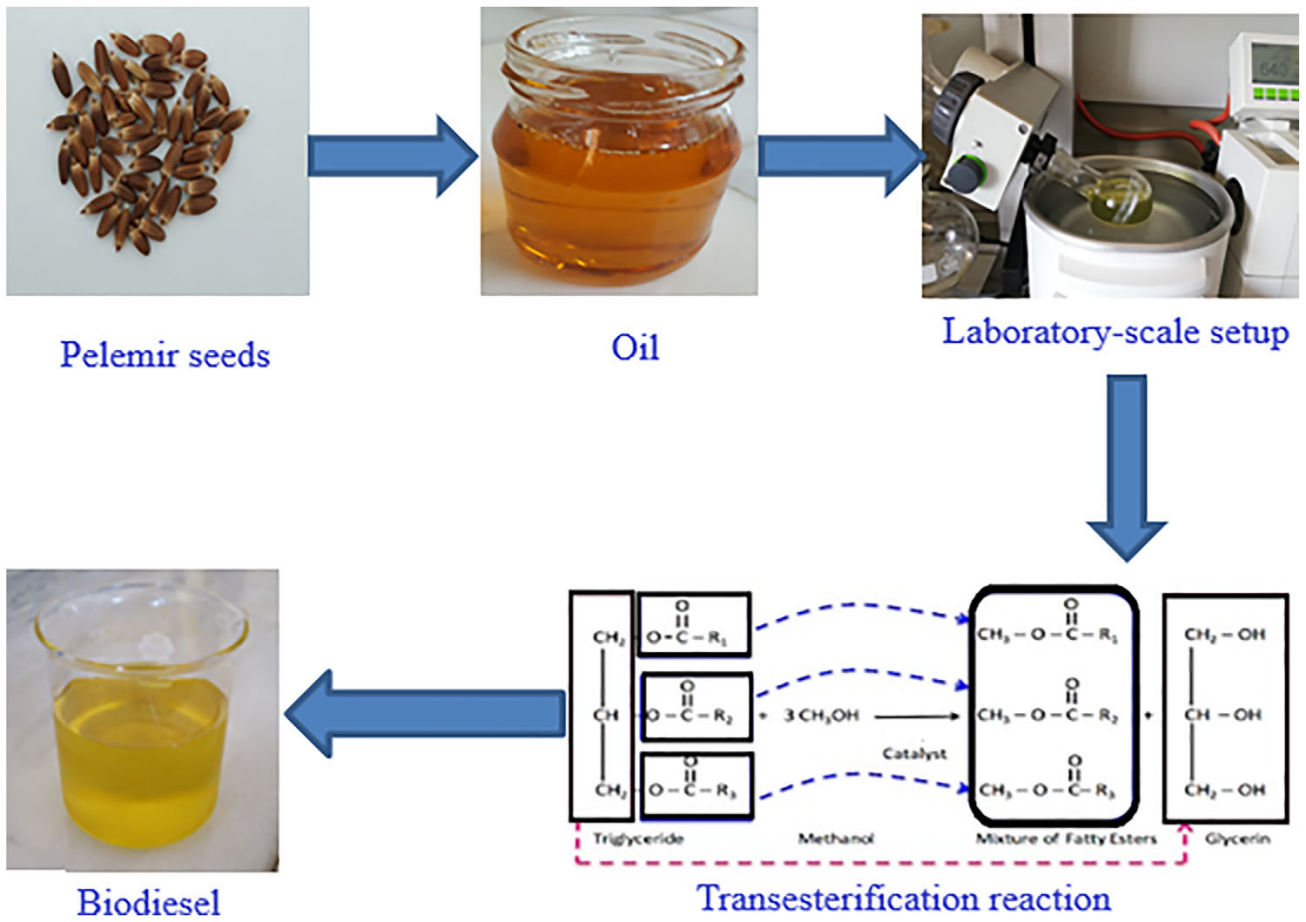
Picture of pelemir seed oil biodiesel.

In this study, the transesterification reaction of crude pelemir seed oil with methanol via base potassium hydroxide catalysis was carried out in a laboratory‐scale setup. The transesterification reaction was performed in a lab‐scale rotary evaporator consisted of a 1 L glass flask. Since the free fatty acid value of pelemir seed oil was below 1%, methyl ester was obtained by using the transesterification method in the presence of a basic catalyst. In this process, methyl alcohol was used 99% purity. In the first step, crude pelemir oil was placed in a glass flask and heated to 30°C. Before the transesterification process, preliminary experimental studies were carried out to determine the optimal process parameter (alcohol:oil molar ratio, reaction temperature, catalyst concentration, and reaction time) levels that would yield the maximum methyl ester production. Transesterification was performed at an 8:1 alcohol:oil molar ratio, a reaction temperature of 50°C, 1% (w/w_oil_) KOH catalyst concentration, and a 60‐min reaction time. The specified amount of catalyst was completely resolved with methyl alcohol and poured onto the heated oil in the flask of the rotary evaporator, and the transesterification was performed at the selected reaction temperature and time. The mixture was stirred at 600 rpm for an hour, and then it was taken into the separatory funnel. After the separation of ester and glycerin occurred during the separation process, the glycerin collected in the lower phase at the bottom of the separatory funnel was removed. The remaining methyl ester was washed repeatedly with distilled water to remove any remaining impurities. After resting for a while, the methyl ester of pelemir seed oil was transferred to the evaporator to remove any remaining water and alcohol. In the final stage, the small particles suspended in the PSOME were separated in a centrifuge at 4000 rpm and 3600 RFC (Relative Centrifugal Force) for 40 min. The resulting methyl ester was stored in glass storage containers. Figure 3 shows an illustration of PSOME production in this study.

Finally, to determine the percentage of conversion of crude oil to biodiesel, the sample was injected into the GC system, and then the biodiesel conversion rate was calculated according to the graph extracted from the GC. Equation (1) was used to calculate the biodiesel conversion rate [7, 46].

(1) $$\mathrm{PSOME}\;\mathrm{Yield}\;\left(\%\right)=\frac{\mathrm{weight}\;\mathrm{of}\;\mathrm{PSO}\;\mathrm{biodiesel}\;\mathrm{produced}}{\mathrm{weight}\;\mathrm{of}\;\mathrm{PSO}\;\mathrm{used}\;\mathrm{in}\;\mathrm{reaction}}\;\times 100$$

#### 2.2.3 Pelemir Seed Oil (PSO) Fatty Acid Profile by GC and Physico‐Chemical Properties

Most of the analyses were carried out in the laboratories of DB Agricultural Energy company in Izmir, Türkiye. Fatty acid composition of crude PSO was determined by using an Agilent 8890 GC gas chromatogram. The Agilent 5977C GC/MSD was used with hydrogen carrier gas and a 20 m long column. The inside diameter is 0.18 mm ID, and the film thickness is 0.25 µm. The detector is FID. The temperature of the GC injector is 250°C. Split ratio is 51.6, pressure 150 kPa, total flow 224.6 mL/min. The flame ionization detector (FID) temperature is 150°C. The oven temperature is kept at 80°C for 10 min. The oven temperature is constant for 14 min. Prior to transesterification, oil physico‐chemical properties of the crude PSO were determined. These physico‐chemical properties included percentage oil content, color, acid and free fatty acid value, crude fiber, ash, protein, water, specific gravity, pH, refractive index, saponification number, unsaponifiable substances, iodine number, freezing point, flash point and peroxide value.

#### 2.2.4 PSOME Fatty Acid Profile by GC

A gas chromatography method is used for the simultaneous determination of mono‐, di‐, and tri‐glycerides in vegetable oil methyl esters. Analyses were performed at the Fuel Research Laboratory of TUBITAK Marmara Research Centre, Gebze, Türkiye. The PSOME was analyzed by the Agilent Technologies GC equipped with a 6890N Network gas chromatogram system. The column is Agilent DB‐Wax (30 m × 0.32 mm × 0.25 µm), with the injector temperature of 250°C and the injector vol ume of 1 µl. The carrier gas is helium at a constant flow rate of 25 mL/min. The flame ionization detector is at 250°C. The oven temperature is initially 200°C and is maintained at 200°C, for 12 min, then increased by 5°C/min until 230°C, and maintained at 230°C for 10 min.

#### 2.2.5 Fuel Properties of PSOME

The PSOME sample produced with the conditions of 8:1 molar ratio of alcohol, 1% (w/w_oil_) KOH catalyst, 1 h reaction time, and 50°C reaction temperature was analysed at the Fuel Research Laboratory of TUBITAK Marmara Research Centre and DB Agricultural Energy Laboratory. The fuel properties of the PSOME produced were determined following EN 14214 and ASTMD 6751 specifications. Determinations of ester content, linolenic acid methyl ester, poly unsaturated methyl ester, carbon residue, density, kinematic viscosity, copper band corrosion, cold filter plugging point, water content, iodine value, total contamination, methanol content, mono‐, di‐ and tri‐glycerides content, free and total glycerol, phosphorus content, sulfur content, acid value, flash point, heating value, oxidation stability and sulfated ash content made.

## 3 Results and Discussion

### 3.1 Characterization of the Crude Pelemir Seed Oil

Vegetable oils consist of a variety of saturated and unsaturated fatty acids. Table 1 provides a comparison of the properties of crude oil obtained from pelemir seeds grown in the Erzincan region and those of pelemir seed oils from different regions of Türkiye in the literature.

**TABLE 1:** Fatty acid composition of pelemir seed oil [32, 35, 47, 48, 49, 50].

Fatty acid	PSO (Present study) %	Yazıcıoğlu (1973)	Yazıcıoğlu (1974)	Yazıcıoğlu et al. (1977)	Altıniğne (1985)	Öğüt et al. (2014)	Duman (2023)
Lauric acid (C12:0)	0.775	1.35	1.62	1.48	1.16	1.717	—
Myristic acid (C14:0)	13.677	18.39	20.50	19.50	16.14	23.44	21.06
Palmitic acid (C16:0)	8.72	8.82	9.96	9.4	9.61	—	10.80
Stearic acid (C18:0)	2.22	1.86	2.03	2	1.93	—	2.26
Oleic acid (C18:1)	27.79	33.33	28.37	30.8	30.98	—	29.17
Linoleic acid (C18:2)	33.9	36.25	37.52	36.9	12.07	41.076	35.56
Linolenic acid (C18:3)	0.416	—	—	—	—	—	0.17
Erucic acid (C22:1)	12.12	—	—	—	—	—	0.03
Palmitoleic acid (C16:1)	—	—	—	—	—	—	0.28
Arachidic acid (C20:0)	—	—	—	—	—	—	0.32
Pentadecanoic acid (C15:0)	—	—	—	—	—	11.858	—
Heptadecanoic acid (C17:1)	—	—	—	—	—	3.0181	—
Caproic acid (C6:0)	—	—	—	—	—	0.0039	—
Elaidic acid (C18:1n9t)	—	—	—	—	—	1.3484	—
gamma‐linolenic acid (C18:3n6)	—	—	—	—	—	0.3133	—
Docosahexaenoic acid (C22:6n3)	—	—	—	—	—	16.6256	—
Epoxy acid	—	—	—	7.77	—	—	—
Monoepoxy fatty acid	—	—	—	—	6.08	—	—
Diepoxy fatty acid	—	—	—	—	4.54	—	—
Unsaturated‐ὰ.ᵝ‐ keto fatty acid					12.97		

Fatty acid analysis revealed that pelemir seed oil obtained from the Karahan genotype contained 25.392% saturated and 74.226% unsaturated fatty acids. Among the unsaturated fatty acids, linoleic acid had the highest percentage at 33.9%, followed by oleic acid at 27.79%. Among the saturated fatty acids, myristic acid had the highest percentage at 13.677%, followed by palmitic acid at 8.87%. Using the fatty acid composition results, the molecular weight of pelemir seed oil was calculated for use in the transesterification process.

Table 1 shows that when compared with the fatty acid compositions determined in studies in the literature, the oleic, lauric, palmitic, myristic, and linoleic acid values of PSO produced from the Karahan genotype are slightly lower. In the literature, the lowest linoleic acid value was measured in the study by [50]. Unlike other studies, 12.12% erucic acid was detected in the oil composition in this study. Erucic acid (cis‐13‐dococenoic acid) is a 22‐carbon monounsaturated fatty acid. Found in high concentrations in the seeds of plants belonging to the Brassicaceae family, including rapeseed and mustard, this fatty acid is a typical component of mustard and rapeseed. Jojoba oil also contains 10%–20% erucic acid. Currently, erucic acid is classified as a natural toxin in the food regulations of many countries, and its potential presence in certain foods is restricted. The Turkish Food Codex Contaminants Regulation sets the maximum limit for erucic acid at 5% of total fatty acids in oils and foods with more than 5% added fat, and 10% in other foods where it may be present [51]. Jojoba oil, with its high erucic acid content and resistance to high temperatures and pressures, is a sought‐after vegetable oil in the automotive and metallurgy industries [52, 53]. The seeds of rapeseed, jojoba, and mustard plants, which contain high levels of erucic acid, are cultivated for use as raw materials in industries other than food, and especially in biodiesel production. This situation supports the suitability of the Pelemir plant variety used in our study as a biofuel feedstock.

### 3.2 Physico‐Chemical Properties of Crude Pelemir Seed Oil

Characterizing vegetable oils is essential for evaluating their applicability in biodiesel production. The fuel properties of biodiesel are characterized by their physicochemical properties. The properties of biodiesel depend primarily on the type of feedstock and its fatty acid composition. The physicochemical quality parameters such as oil content, color, acid value, free fatty acid value, protein, water, crude fiber, ash, specific gravity, kinematic viscosity, pH, refractive index, saponification number, unsaponifiable substances, iodine value, freezing point, flash point, peroxide value and oxidative stability of the crude pelemir seed oil were determined by using AOCS Tentative Methods [54]. The physicochemical properties of crude PSO extracted under laboratory conditions were listed in Table 2 along with the results of similar studies in the literature. In the present study, the physicochemical properties of PSO are similar to the measurement results obtained in other studies in the literature, as shown in Table 2.

**TABLE 2:** Physico‐chemical quality parameters of crude oil from pelemir seeds [32, 35, 47, 48, 49].

Parameters	Present study	Yazıcıoğlu 1951	Yazıcıoğlu 1973	Yazıcıoğlu 1974	Yazıcıoğlu et al., 1977	Öğüt et al., 2014	Duman, 2023
Oil content %	23.19	24.8	24.9	25.8	25.3	22.40	20.91
Color	5,77L‐5,12a‐8,16b	—	—	—	—	—	2.8 R‐ 70 Y‐0B ‐
Acid value (mg KOH/g)	0.96	—	—	—	—	—	0.40
Free fatty acid value (% oleic acid)	0.48	0.6	0.21	0.24	0.23	—	—
Protein, %	17.66	17.0	14.8	16.9	15.9	—	—
Water, %	7.03	6.1	6.4	9.2	7.8	—	—
Crude fiber, %	11.2	10.4	11.9	11.8	11.9	—	—
Ash,%	6.9	5.72	7.80	5.30	6.5	—	—
Specific gravity (25°C, g/cm^3^)	0.926	0.9263	0.9241	0.9217	0.9229	—	0.925
Kinematic viscosity (mm^2^/s)	13.2						
Dynamic viscosity (20°C) (cP)	—	84	—	—	84	—	84.40
Ph	5.26	—	—	—	—		—
Refractive index (25°C)	1.468	1.4716	1.4702	1.4711	1.4706	—	1.4708
Saponification number (mg KOH/g)	191.8	194.9	190.0	193.6	192	—	196.98
Unsaponifiable substances (g/100 g)	1.23	1.32	1.28	1.19	1.24	—	1.05
Iodine value	94.9	85.8	89	87.8	88.4	—	89.45
Rodan index	—	—	—	—	58.8	—	—
Reichert‐Meissl index	—	0.25	0.33	0.38	0.36	—	—
Polenske index	—	0.15	—	—	0.25	—	—
Hydroxyl index	—	20	22.2	19.7	20.9	—	—
Tiyosiyonojen index	—	65.0	58.4	59.2	—	—	—
Freezing point	−16	‐ 15	—	—	—	—	—
Flash point (°C)	235	—	—	—	—	—	238
Vitamin E (mg/100 g)	—	—	—	—	—	—	51.90
Peroxide value(%)	4.82	—	—	—	—	—	4.93
Oxidative stability (h)	2.45	—	—	—	—	—	2.32

When considering PSO as a feedstock source, especially in biodiesel production, its oil content, free fatty acid value, specific gravity, kinematic viscosity, iodine value, freezing point, and flash point values stand out.

When the table is examined, similar studies in the literature show that the oil content of the pelemir plant varies between 20.91% and 25.8%. In this study, the oil content of the plant was measured as 23.19%. These differences can be observed due to the inverse relationship between yield and oil content in oilseeds and changes in environmental conditions. Seed yield per acre increases, especially during growing seasons with sufficient rainfall.

The table shows that studies examining pelemir seed oils from different regions of Türkiye exhibited FFA content ranging from 0.21% to 0.6%. Our study's FFA content of 0.48% is consistent with these studies. Water and free fatty acids lead to saponification reactions with the basic catalyst present in the environment during the transesterification reaction, which causes significant problems in biodiesel production. In basic biodiesel production, keeping the FFA below 1% is among the most important parameters for methyl ester efficiency [55, 56, 57]. The PSO specific gravity value is similar to the results reported in the literature.

The iodine value in vegetable oils is not constant. It is affected by the location where the plant is grown, the maturity of the seed, and climatic conditions. PSO's low iodine value, consistent with the literature, makes it more advantageous than many other oilseeds in biodiesel production. Since PSO also has an iodine value below 100, it belongs to the group of non‐drying oils, like jojoba oil [58].

PSO has a low freezing point, allowing it to operate in cold weather conditions, and its high flash point ensures safe handling and storage. In addition to its potential as a fuel, the seed meal remaining after oil extraction is also promising for animal feed because it is rich in protein and fiber.

### 3.3 Fuel Properties of Biodiesel From Pelemir Seed Oil

Since biodiesel is produced from vegetable oils of varying origin and quality in plants of different sizes, standardization of fuel quality is necessary to ensure the most efficient and continuous engine performance. In this study, the fuel properties of the methyl ester of pelemir oil, which we produced under optimum conditions, were shown in Table 3, compared with the international standards EN 14214 and ASTM D 6751.

**TABLE 3:** Fuel properties of PSOME.

Analys	Unit	Method	PSOME	EN 14214		ASTM D 6751
				Low	Upper	
Ester content	%(m/m)	EN 14103	96.5	96.5	—	
Linolenic acid methyl ester	%(m/m)	EN 14103	1.58	—	12	
Poly unsaturated methyl ester	%(m/m)	EN 14103	0	—	1	
Carbon residue	%(m/m)	EN ISO 10370	0.18	—	0.30	
Density, 15 ^o^C	g/cm^3^	EN ISO 3675	0.87	0.86	0.90	No specified limit
Kinematic viscosity	mm^2^/s	EN ISO 3104	4.36	3.5	5	1.9 – 6.0
Copper band corrosion (3 h at 50°C)	—	EN ISO 2160	1a	—	1	max No.3
Cold filter plugging point	^o^C	EN 116	−9	+5(Summer)‐15(Winter)		No specified limit
Water content	mg/kg	ENISO12937	210	—	500	max 500
Iodine value	g iodine/100 g	EN 14111	94.9	—	120	
Total contamination	mg/kg	EN 12662	18	—	24	
Methanol content	%(m/m)	EN 14110	0.03	—	0.20	
Monoglyceride content	%(m/m)	EN 14105	0.4	—	0.80	
Diglyceride content	%(m/m)	EN 14105	0.07	—	0.20	
Triglyceride content	%(m/m)	EN 14105	0.06	—	0.20	
Serbest glycerol	%(m/m)	EN 14105	0.008	—	0.02	max 0.02
Total glycerol	%(m/m)	EN 14105	0.12	—	0.25	max 0.24
Phosphorus content	mg/kg	EN 14107	3.8	—	4	max 10ppm
Sulfur content	mg/kg	EN ISO 20846	0	—	10	
Acid value	mgKOH/g	EN 14104	0.46	—	0.50	max 0.5
Flash point	^o^C	ASTM D93	230	120	—	min 93
Heating value	Mj/kg	ASTM D 240	39.25	35	—	No specified limit
Oxidation stability,110°C	Hour	EN 14112	7.8	6	—	min 3
Sulfated ash content	%(m/m)	EN ISO 3987	0.01	—	0.02	max 0.02
Sulfur content	mg/kg	EN ISO 20846	0	—	10	

Fatty acid methyl esters are formed as a result of the reaction of oils or fatty acids with methanol, and they differ in terms of the chain length and number of double bonds of the fatty acids. The content of fatty acid methyl esters is called ester content and is a measure of the purity of FAME. The EN 14214 biodiesel standard requires a minimum methyl ester content of 96.5% (m/m). The fact that a conversion efficiency of 96.5% (at 50°C, 60 min, 8:1 alcohol:oil molar ratio and 1% (w/woil) KOH catalyst concentration) is achieved in the transesterification of pelemir oil using the catalytic method makes the pelemir plant an economically attractive feedstock source. It is one of the clean and carbon cycle‐neutral fuels.

Properties of biodiesel, such as its linolenic acid and polyunsaturated methyl ester content, are closely related to the fatty acid profile of PSO. In this study, these properties of PSOME remain well below the upper limit values specified in the EN14214 standard. Therefore, the fatty acid profile of PSO is suitable for methyl ester production.

Density and viscosity values in biodiesel fuel are factors that have a significant impact on engine performance. Another important characteristic of biofuel is its density. Relatively low density means lower fuel consumption and less particulate matter, unburned hydrocarbon, and carbon dioxide emissions. According to the EN 14214 biodiesel standard, the density of the fuel at 15°C should be between 0.86 and 0.90 g/cm^3^, while the ASTM D 6751 standard requires values between 0.82 and 0.90 g/cm^3^ [18, 59, 60, 61]. This property is particularly important in anaerobic combustion systems because the density of the fuel directly affects atomization efficiency. In this study, the density of biodiesel produced from crude pelemir oil was measured as 0.87 g/cm^3^, which means it is suitable of standard.

Kinematic viscosity is an important parameter in diesel engines for fuel atomization, efficient combustion, and enough to lubricate. Viscosity below or above standard values creates problems with injection performance in the injector. Low viscosity indicates the presence of high concentrations of monounsaturated fatty acid methyl esters. The low viscosity of biodiesel fuel helps improve atomization and combustion efficiency in diesel engines. Issues such as higher viscosity and lower oxidation stability can lead to problems with engine performance, especially in cold climates [55, 56, 59, 62]. For biodiesel to be used in diesel engines, its kinematic viscosity must be between 3.5 and 5.0 mm^2^/s according to EN 14214 standards. In this study, the PSOME kinematic viscosity was measured as 4.36 that means it is suitable of standard.

Cold Filter Plugging Point (CFPP) is a critical parameter that determines the lowest temperature at which a given volume of fuel can pass through a standard filtering device. This parameter is important for preventing fuel filter clogging and engine problems in cold weather conditions [6, 63]. The low‐temperature performance of biodiesel largely depends on its molecular structure, nature of feedstock, and its degree of saturation. For PSOME, the cold filter plugging point was measured at 50°C, indicating that this biofuel can be used even in subtropical regions.

The iodine value is a used parameter that measures the degree of unsaturation in oils and fats. Oils rich in saturated fatty acids have a low iodine number. The iodine content varies depending on the type of feedstock used. Since unsaturated fatty acids are more susceptible to oxidation, biodiesel with a high iodine content is less stable against oxidation. Therefore, the iodine content of biodiesel is considered a stability parameter. Iodine value measures the unsaturation degree of biodiesel and influences its stability during storage and use. EN 14214 specifies a maximum value of 120 g iodine/100 g. In many oil feedstocks, the iodine content of the oil has been found not to meet the standards [3, 60, 64]. The PSOME iodine value was measured as 94.9 g iodine/100 g. This iodine value is lower than that of canola (100–115), soybean (125–138), safflower (138–151), and corn (110–128), which are used as feedstock in biodiesel production both in the world and in our country [65, 66]. PSOME, with its low iodine content, tends to be more saturated and stable, making it safe to use as fuel in diesel engines.

The transesterification of vegetable oils with methanol is an equilibrium reaction. The main product in this reaction is FAME. Depending on the reaction conditions, the end product consists of the intermediate phases of the reaction (mono‐ and diglycerides) and unconverted vegetable oils (triglycerides). During the transesterification process, free glycerol is released from the oil and fats. Oxidation stability is the tendency of fuels to react with oxygen at temperatures close to ambient temperature. The oxidation of biodiesel results from a chemical change that occurs after contact with air [61, 63]. In this study, PSOME's oxidation stability was 7.8 h, which meets the standards. In this form, the fuel is also suitable for long‐term storage without deterioration.

Acidity is a measure of the acid content of biodiesel and its potential corrosiveness. During the transesterification process, the reaction of free fatty acids from the feedstock with a catalyst results in the formation of alkaline soaps. These soaps are largely removed from the product through physical separation, but the remaining soap residues are broken down by washing with inorganic acids, and the resulting free fatty acids remain in the biodiesel as a fat‐soluble component [67, 68]. The standards limit the acid value to 0.5 mg KOH/g, which corresponds to approximately 0.25% fatty acid content and prevents biodiesel from being corroded by acids. In PSOME, this value was measured as 0.46 mg KOH/g. The corrosive properties of the fuel comply with standards.

High monoglyceride content in oils can lead to coking and deposit accumulation in the injection systems of diesel engines. Filter clogging can occur, especially during operation in cold weather conditions [55, 62, 63]. The limit value for monoglycerides in the EN 14214 standard is 0.80% (m/m). In this study, the monoglyceride content of PSOME is 0.40% (m/m). It is significantly lower than the standard. This allows the fuel to be used safely, especially in cold weather conditions, without clogging the filters. Similarly, injector systems in diesel engines will be protected against deposits and carbon buildup.

Diglycerides, triglycerides, and free glycerol can cause coke formation in the injection system and cylinders of diesel engines at high temperatures and under conditions of incomplete combustion. Therefore, the maximum content of diglycerides and triglycerides is limited to 0.20% (m/m), and the free glycerol content to 0.02%(m/m) [18, 59, 61]. PSOME's diglyceride, triglyceride, and free glycerol values were measured as 0.07, 0.06, and 0.008%(m/m), respectively. In this study, these values were well below the upper limits. This makes PSOME advantageous as a fuel suitable for combustion.

Phosphorus is found in vegetable oils as phospholipids. It is a typical catalyst poison for exhaust gas aftertreatment systems for diesel engines. Even low phosphorus content can lead to adverse effects in the long term under continuous operating conditions. In biodiesel production from vegetable oils, phosphorus content can be reduced by degumming. Since the flash point is an important criterion for the safety of fuel during storage and transportation, biodiesel produced from pelamir seed oil is also suitable in terms of meeting this criterion [55, 69].

Furthermore, the evaluation of the copper band corrosion parameter is also important and is a qualitative method for determining the corrosiveness of the biodiesel. Ash content can be correlated with the presence of inorganic contaminants such as catalyst residues. These substances cause damage by forming deposits in injectors and the fuel system [14, 46]. PSOME is sulfur‐free. Furthermore, other measured properties of biodiesel produced from pelemir seed oil, such as water content, methanol content, and heating value, also meet the quality requirements according to the international standards EN 14214 and ASTM D 6751.

## 4 Conclusions

In our country, the goal is to reduce the emission of gases that cause global warming into the atmosphere and to achieve net zero emissions by 2053. One of the most fundamental activities to achieve this goal is to diversify energy sources and take steps toward the use of renewable energies. Viewing the world as humanity's common heritage and a shared value, Türkiye is diversifying its energy sources for both humanitarian and strategic reasons. The problem of uninterrupted feedstock supply, which constitutes the largest part of biodiesel production costs, especially in developing countries, can be reduced through the production of nonedible vegetable oil feedstock that do not threaten the food sector. The pelemir plant examined in this study stands out as a potential feedstock source for biodiesel production, both in terms of oil yield and biodiesel fuel properties, without negatively impacting global food sustainability. The characteristics of pelemir oil are very similar to those of diesel fuel. The properties of pelemir biodiesel meet the EN 14214 and ASTM D 6751 biodiesel standards.

Pelemir is a sustainable alternative for agricultural production under climate change pressure, owing to its ability to utilize marginal lands and its tolerance to drought and low temperatures. The fact that the pelemir plant grows in barren areas, making previously unusable land available and creating job opportunities in rural areas, is of great importance in terms of regional development and preventing migration to large cities. It also represents a suitable alternative energy crop for low‐income farmers in rural areas. In particular, cereal growers can cultivate and harvest pelemir without the need for additional equipment. Because its cultivation does not require fertilizers, herbicides, or pesticides, input costs can be substantially reduced, thereby contributing to increased farm income. Thanks to its cold‐ and drought‐tolerant characteristics, pelemir exhibits high ecological adaptability and is less affected by adverse weather conditions associated with climate change.

Using the pelemir plant as a fuel feedstock enables the development of local biofuel supply chains without occupying productive agricultural land needed for food production, thereby contributing to both energy security and increased food resilience in vulnerable regions. Beyond its socio‐economic benefits and compliance with fuel standards, biodiesel derived from pelemir seeds offers significant environmental advantages, particularly in the context of mitigating climate change. Although detailed life cycle assessments are necessary to accurately determine its carbon footprint, the renewable nature of palm oil and its lack of competition with food make it a promising feedstock for a low‐carbon energy future.

Pelemir, which is well suited for cultivation in Türkiye and in regions with similar arid to semi‐arid climates, may help eliminate feedstock supply constraints and thereby support the uninterrupted operation of biodiesel production facilities. In doing so, biodiesel plants can remain competitive while also contributing to the global transition toward cleaner energy systems.

## Conflicts of Interest

The authors declare no conflicts of interest.

## Data Availability

The data that support the findings of this study are available on request from the corresponding author. The data are not publicly available due to privacy or ethical restrictions.
